# Effect of ABA Pre-Treatment on Rice Plant Transcriptome Response to Multiple Abiotic Stress

**DOI:** 10.3390/biom13101554

**Published:** 2023-10-20

**Authors:** Fatemeh Habibpourmehraban, Farhad Masoomi-Aladizgeh, Paul A. Haynes

**Affiliations:** 1 School of Natural Sciences, Macquarie University, North Ryde, NSW 2109, Australia; fatemeh.habibpour-mehraban@hdr.mq.edu.au (F.H.); farhad.masoomi-aladizgeh@mq.edu.au (F.M.-A.); 2 Biomolecular Discovery Research Centre, Macquarie University, North Ryde, NSW 2109, Australia

**Keywords:** rice, multiple abiotic stress, transcriptomics, ABA pre-treatment, photosynthesis

## Abstract

Half of the world’s population depends on rice plant cultivation, yet environmental stresses continue to substantially impact the production of one of our most valuable staple foods. The aim of this study was to investigate the changes in the transcriptome of the IAC1131 rice genotype when exposed to a suite of multiple abiotic stresses, either with or without pre-treatment with the plant hormone ABA (Abscisic acid). Four groups of IAC1131 rice plants were grown including control plants incubated with ABA, non-ABA-incubated control plants, stressed plants incubated with ABA, and non-ABA-incubated stressed plants, with leaf samples harvested after 0 days (control) and 4 days (stressed). We found that high concentrations of ABA applied exogenously to the control plants under normal conditions did not alter the IAC1131 transcriptome profile significantly. The observed changes in the transcriptome of the IAC1131 plants in response to multiple abiotic stress were made even more pronounced by ABA pre-treatment, which induced the upregulation of a significant number of additional genes. Although ABA application impacted the plant transcriptome, multiple abiotic stress was the dominant factor in modifying gene expression in the IAC1131 plants. Exogenous ABA application may mitigate the effects of stress through ABA-dependent signalling pathways related to biological photosynthesis functions. Pre-treatment with ABA alters the photosynthesis function negatively by reducing stomatal conductance, therefore helping plants to conserve the energy required for survival under unfavourable environmental conditions.

## 1. Introduction

Rice is an economically important cereal crop, the productivity of which is severely threatened by diverse environmental stresses, even though it plays a vital role in feeding the world population [1]. With climate change and the constant pressure of environmental disasters and more extreme weather events occurring, there is an urgent need to develop crop varieties that can better tolerate multiple stresses [2]. Furthermore, the immobile nature of plants increases the likelihood that plants experience multiple stresses in the field simultaneously during their lifecycle.

Abiotic and biotic stresses severely affect the growth and reproduction of plants and crops. Rice plants are sensitive at the vegetative growth stage, exposing them to unfavourable conditions causing serious problems [3]. Drought stress is the most serious issue for rice cultivation in rain-fed ecosystems [4], while flooding, salinity, and heat stress can all cause large losses in crop yields and pose serious threats to rice production [5].

The detailed mechanisms of how rice plants deal with multiple abiotic stresses simultaneously remain largely unknown [6]. Elucidation of the exogenous components affecting stress sensitivity or tolerance is another key factor in the development and breeding of rice genotypes with improved productivity and efficacy.

Determining the critical molecular mechanisms and cellular processes responsive to stresses will provide biological insights and information that are relevant for addressing both climate change and food crises. As sessile organisms, plants have evolved distinct strategies to respond and adapt to adverse environmental cues through diverse mechanisms. Changes in metabolic activity and gene expression produce both general and stress-specific molecular responses that help the plant acclimate to changes in the environment [7]. Examples of this include the reduced rate of photosynthesis in response to drought stress in tomato plants [8], the increased expression of antioxidant proteins in response to stress in sugar beets [9], and the increase in proline content and antioxidant activity in apples and cherries in response to osmotic stress [10]. Regulation of gene expression in plants relies on a variety of molecular mechanisms that affect different steps in the lifecycle of a gene, including transcription, splicing, processing, transport from the nucleus to the cytoplasm, translation, and storage [11].

Many plant molecular responses, including the transcriptome profile, are coordinated by phytohormones. The production of phytohormones is altered in response to the imposition of abiotic stresses and, in turn, can also mediate stress responses through the actions of hormone and stress-responsive transcription factors (TFs) [12]. These important signalling molecules can induce stomatal closure, the expression of dehydration tolerance genes, and many other adaptive physiological responses [13]. The functional analysis of TFs in plants can be divided into five general categories: expression analysis, bioinformatic analysis, phenotypic analysis, molecular functional analysis, and network analysis, entailing the characterization of transcriptional regulatory networks [14]. Much of this work has been facilitated by technical innovations in next-generation sequencing, such as RNA-sequencing (RNA-seq), which have accelerated the functional elucidation of genes at the transcriptional, post-transcriptional, post-translational, and epigenetic levels, and therefore have been applied extensively in plant stress research [15].

In this study, transcripts in leaf tissue related to multiple abiotic stress responses and interaction with ABA pre-treatment were investigated using RNA-Seq data analysis for the IAC1131 rice genotype. IAC1131 is a drought-tolerant upland rice cultivar that has been previously shown to be more drought tolerant [16,17], and more tolerant to combined drought, salt, and temperature stress, than Nipponbare, which is a lowland rice that is sensitive to drought and heat stress [3]. Previous studies investigating plant stress responses at the transcriptome level are typically focused on one stress at a time; examples include the transcriptomic characterisation of salt tolerance in germinating rice [18], drought tolerance in rice [19], and salinity–alkalinity tolerance in rice seedlings [20]. In contrast, in this analysis, we analysed the rice transcriptome to explore how rice regulates responses to multiple abiotic stresses imposed simultaneously with or without prior ABA application. We used RNA-Seq to develop a picture of the ABA-dependent and ABA-independent mechanisms and pathways that respond to multiple abiotic stress in rice leaves. The outputs from this study are candidate genes associated with ABA signalling and multiple stress responses in plants, which represent multiple stress biomarkers that are interesting and valuable candidates for further study.

## 2. Materials and Methods

### 2.1. Plant Material and Stress Treatment

Rice seeds were sterilized prior to sowing using four washing steps: 70% ethanol for 20 min; water for 1 min; 50% bleach solution for 30 min; and water for 5 min. A soil mixture was prepared, consisting of 35% (*v*/*v*) peat mix, 25% (*v*/*v*) peat moss, 25% (*v*/*v*) Waikerie river sand, and 15% (*v*/*v*) clay loam, supplemented with NPK 23:4:14 fertiliser. The greenhouse used for growing plants was set to a 12-h photoperiod with a light intensity minimum of 700 µmolm^−2^s^−1^ and day/night temperatures of 28/22 °C. Five seeds of IAC1131 rice (*Oryza sativa*) genotype were initially sown in 30 cm deep, 10 cm-diameter pots containing 700 g of soil. The most vigorous seedlings were selected to be grown for further study. Additional NPK fertiliser was applied to the soil after 2 weeks of growth. There were 12 pots in total used for sample collection in this study each containing one plant, constituting three replicates each of all four treatment regimes applied. The plants were grown in a greenhouse under controlled conditions with the temperature set to 28/22 °C (day/night), a 12-h photoperiod, and the light intensity set to a minimum of 700 µmolm^−2^s^−1^.

Seedling plants were grown to the vegetative stage. After three weeks of additional growth, the plants were separated into four groups, each one comprising three plants in individual pots. The first group was watered every day up to full field capacity (100%) as previously determined by weighing pots to measure daily water loss, and sprayed with distilled water. These were not subject to stress and were not pretreated with ABA and hence were labelled as (Control − ABA) plants. The second group were also kept under well-watered conditions in which the plants were watered every day up to full field capacity and were sprayed with 100 µM of ABA hormone for 2 days consistently and labelled as (Control + ABA) plants. The third group was kept under well-watered conditions and sprayed with distilled water prior to the imposition of the multiple abiotic stress treatment for 4 days and labelled as (Stress − ABA). The last group of plants was sprayed with 100 µM ABA hormone for 2 days consistently prior to the imposition of the multiple abiotic stress treatment for 4 days and labelled as (Stress + ABA).

Multiple abiotic stress was imposed on the plants after a 2-day period of ABA or distilled water spraying, and a 2-day stress pre-treatment. The multiple simultaneous abiotic stresses that were applied consisted of 50 mM NaCl as salt stress, 50% field capacity watering as drought stress, and 30/18 °C day/night temperature as temperature stress. In order to generate simultaneous drought and salt stresses, 2 days of stress pre-treatment was applied in which the plants were watered with 25 mM NaCl to reach 100% field capacity, then watering was stopped until the field capacity reduced to 50%, producing a final NaCl concentration of 50 mmol. Transpiration of water from the plants was recorded daily by weighing the pots and the multiple abiotic stress treatment commenced with the application of a temperature stress of 30/18 °C (day/night). After 0 days (control) and 4 days (stress), the 5 youngest, fully expanded, fresh leaves were used for measuring physiological parameters, then collected as three biological replicates and immediately lyophilized in liquid nitrogen. For further analysis, frozen leaf samples were placed in 2 mL centrifuge tubes and finely ground using a TissueLyser II (Qiagen Retsch 12090, Qiagen, Hilden, Germany), with liquid nitrogen and five (2.8–3.3 mm) Zironox beads.

### 2.2. Physiological Measurements

At time points representing zero days and four days of exposure to multiple abiotic stress, the measurement of gaseous CO_2_ exchange parameters was performed for both the ABA-incubated and non-ABA-incubated plant groups. Measurements taken included stomatal conductance (gs) and the ratio of intercellular to ambient CO_2_ concentration (Ci/Ca). Photosynthesis variables were recorded using a LICOR photosynthesis system (LI-6400, LI-COR, Inc., Lincoln, NE, USA), measuring the 5 youngest, fully expanded leaves at midday for each treatment from 3 biological replicate plants. Parameters employed included a CO_2_ concentration of 400 µmolm^−1^, relative humidity at 50%, the temperature of leaf chamber being adjusted to 30 °C and 33 °C for the control and stressed plants, and a photosynthesis active radiation at 1800 µmolm^−2^s^−1^. Results are presented as the mean value means ± standard error from the analysis of the three biological replicates.

### 2.3. RNA Isolation

An RNeasy Plant Mini Kit (Qiagen, Hilden, Germany) was used to extract total RNA from rice leaf samples. In brief, leaf tissue was finely ground and 500 μL of the extraction buffer and 25 μL of beta-mercaptoethanol were added prior to the samples being incubated at 65 °C for 5 min. The mixture was vigorously vortexed and then centrifuged for 5 min at 34,000× *g* at 4 °C. Subsequently, the 450 µL of supernatant (450 μL) was transferred into a QIAshredder Mini spin column and RNA was isolated according to the manufacturer’s instructions. The purity, integrity, and concentration of the prepared RNA were assessed using a TapeStation (Agilent Technologies, Santa Clara, CA, USA) and an Epoch Take3 spectrophotometer (BioTek, Winooski, VT, USA).

### 2.4. Library Preparation and Illumina Sequencing

The preparation of the RNA and Illumina sequencing RNA libraries were both performed at the Ramaciotti Centre for Genomics (Sydney, NSW, Australia), using a QIAseq Stranded Total RNA Library Kit (Qiagen, Hilden, Germany) according to the manufacturer’s instructions. All of the individual libraries were pooled together after the initial normalization. Sequence data was then generated on a single flow cell lane of an Illumina NovaSeq 6000 system, producing 75 bp single-end reads, with approximately 30 million reads per sample. The quality control criteria applied was a pass threshold of >80% of bases higher than Q30 at 1 × 75 bp, and a downstream analysis of the data outputs was performed using FASTQ files.

### 2.5. RNA-Seq Data Acquisition and Processing

#### 2.5.1. Quality Check and RNA-Seq Data Analysis

Raw data were checked for quality using FASTQ Quality Check software (Version 0.11.9) [21]. Adapter contamination and low-quality reads were removed using Trimmomatic preprocessing software version 0.40 [22]. *De novo* transcriptome assembly of the resultant high-quality reads was performed using Trinity software (version 2.14) [23]. A quantitative assessment of the assembled transcriptome was performed based on evolutionarily-informed expectations of gene content from Benchmarking Universal Single-Copy Orthologs (BUSCO) selected from OrthoDB [24,25]. Candidate coding regions within transcript sequences were functionally detected using TransDecoder software (version 5.5) [26]. High-throughput sequencing data were mapped to quantify gene expression with RNA-seq alignments using STAR [27], and a rice reference genome sequence (downloaded from NCBI on 30 March 2022, containing 52,629 gene sequences). Read counts were generated from the alignment files to estimate the gene and isoform expression levels from the RNA-Seq data [28]. The number of mapped clean reads for each gene was counted and normalized into the reads per kilobase per million (RPKM) value. Differentially expressed genes (DEGs) were analysed using the edgeR package and Bowtie2 [29]. Threshold levels of FDR  ≤  0.1, Students *t*-test *p*-value < 0.05, and log_2_ FC  >  1 or log_2_ FC  <  − 1, were used to determine the significance of differences.

RNA-seq data have been deposited to the National Centre for Biotechnology Information via the NCBI submission portal with BioProject identifier SUB12326911.

#### 2.5.2. Gene Function Annotation

Gene ontology and pathway enrichment analysis gene ontology (GO) enrichment analysis was performed using agriGO (http://systemsbiology.cpolar.cn/agriGOv2, accessed on 19 June 2022) [30]. GO terms with FDR ≤ 5% were considered to be significantly enriched. The Fisher exact test and Yekutieli multi-test adjustment (FDR under dependency) methods were applied and significant GO terms with *p*-value  <  0.05 were retrieved. All of the corresponding transcripts were also used in searches against the significant threshold e-value  ≤  10^−5 ^ (FDR) [31]. A pathway analysis was performed using KOBAS software (https://bio.tools/kobas, accessed on 14 October 2022) to test the statistical enrichment of DEGs in KEGG pathways [32]; pathways with FDR ≤ 5% were considered to be significantly enriched.

## 3. Results

### 3.1. Experimental Design and Plant Physiology

**Figure 1:**
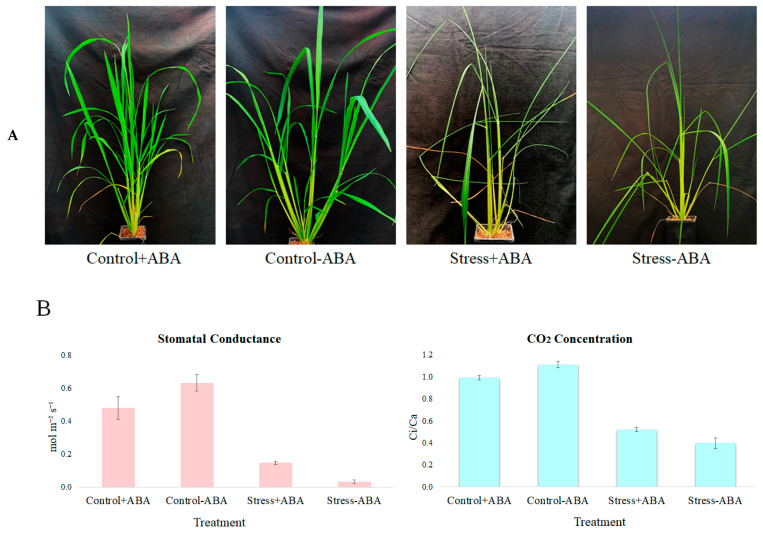
(**A**) IAC1131 plants used in this study grown under control and multiple abiotic stress conditions, incubated and non-incubated with ABA. (**B**) Stomatal conductance and ratio of intracellular to ambient CO_2_ concentration (Ci/Ca); standard errors were obtained from 3 biological replicate measurements.

IAC1131 rice genotype plants were grown to the vegetative stage (Figure 1A) and, after four weeks of growth, four groups of plants, namely (Control − ABA), (Control + ABA), (Stress − ABA), and (Stress + ABA), were used for transcriptome level analysis. Comparative transcriptomics analysis was used to investigate the changes in transcriptome profiles in leaves from the IAC1131 rice plants.

Morphological comparison, stomatal conductance (gs), and the ratio of intercellular to ambient CO_2_ concentration (Ci/Ca), all clearly indicated evidence of stress response in the plants. As shown in Figure 1, the plants subjected to stress were noticeably smaller and less vigorous, and both gs and Ci/Ca were greatly reduced in the stressed plants compared to their unstressed counterparts. Interestingly, the addition of exogenous ABA appeared to have different effects depending on whether the plants were exposed to stress or not. In unstressed plants, the addition of exogenous ABA increased both stomatal conductance and gs, while in plants exposed to stress, the addition of exogenous ABA caused both parameters to decrease significantly.

### 3.2. Transcriptome Profile in Rice Plants Exposed to Multiple Abiotic Stress

Illumina deep sequencing technology was employed to sequence the total RNA from the leaf tissue of three biological replications of the four sample groups of IAC1131 rice plants [(Control − ABA), (Control + ABA), (Stress − ABA), and (Stress + ABA)]. In total, we obtained more than 360 million raw reads with at least 16 million reads for each condition (Table 1). These reads were reconstructed using Trinity software [23] to assemble *de novo* the transcriptome from the RNA sequencing data. The assembly of the sample reads resulted in the identification of a total of 120,071 transcripts and 82,387 genes, based on a reference rice genome sequence from the NCBI submission portal. More than 99% of raw sequences were retained with high quality after adaptor and low-quality sequences were removed by trimming. The assembled high-quality reads from the individual samples of IAC1131 plants mapped against the rice genome ranged from 70% to 95% (Table 1).

**Figure 2:**
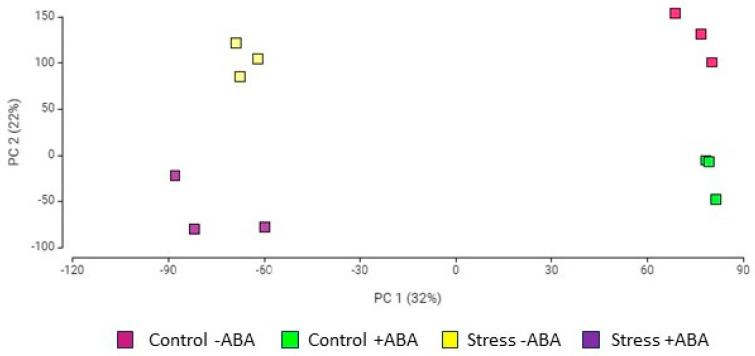
Principal component analysis (PCA) of the transcriptome data from the four groups of IAC1131 plants: (Control − ABA), (Control + ABA), (Stress − ABA), and (Stress + ABA).

Principal component analysis (PCA) was performed on the ABA-incubated and ABA non-incubated samples, before and after multiple abiotic stress treatments. The first component (PC1) and the second component (PC2) explained 32% and 22% of the total variation in RNA count distribution of the four sets of samples (Figure 2). Replicate samples at each condition were highly similar in all cases and transcriptome expression patterns in response to multiple abiotic stress treatments were easily distinguishable from the control plants. Likewise, data from ABA-incubated and non-ABA-incubated plants were clearly separated, indicating that there were obvious dimensional differences between the samples.

To investigate the genes altered in expression in both stress response and ABA signalling, comparative pairwise transcriptome analysis of the rice leaf samples was performed for control and stressed plants, incubated and non-incubated with ABA, which resulted in the identification of differentially expressed genes (DEGs) up- or down-regulated under different conditions.

**Figure 3:**
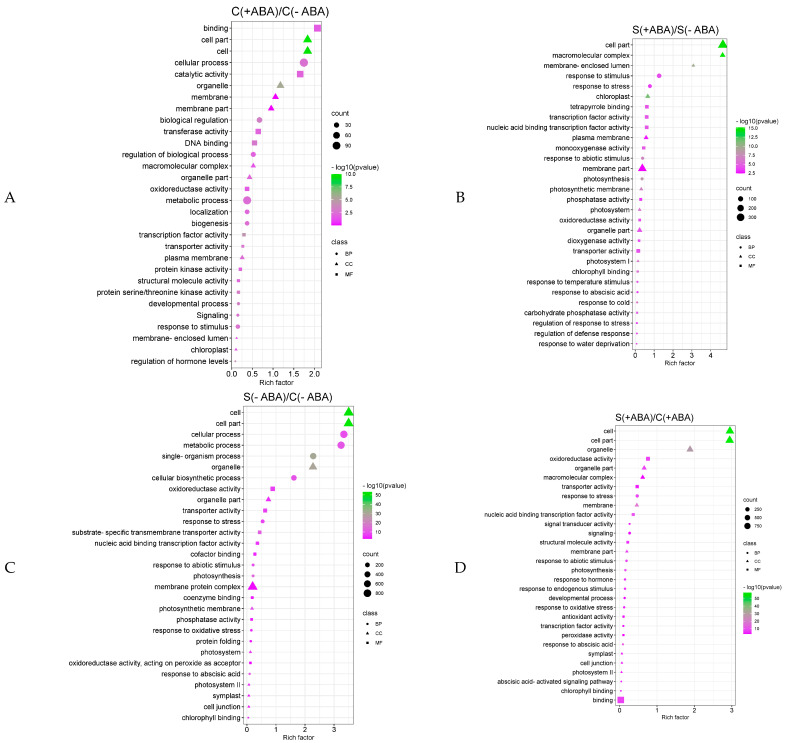
GO enrichment of the DEGs from the four groups of IAC1131 plants. Scatter plot of enriched GO in (**A**) [C(+ABA)/C(−ABA)] comparison; (**B**) [S(+ABA)/S(−ABA)] comparison; (**C**) [S(−ABA)/C(−ABA)] comparison; and (**D**) [S(+ABA)/C(+ABA)] comparison. Rich factor (*x*-axis) is the ratio of the differentially expressed gene number to the total gene number in a given enriched GO category. The color and size of the dots represent the range of the -log *p*-value and the number of DEGs mapped to the indicated enriched GO, respectively.

Functional enrichment analysis revealed significant biological processes (BP), molecular function (MF), and cellular process (CC) in which the multiple abiotic stress, ABA pre-treatment, or their interaction, might participate. Cellular process, biological regulation, and regulation of biological process were the most enriched biological processes in the [C(+ABA)/C(−ABA)] comparison (Figure 3A). Similar analysis for [S(+ABA)/S(−ABA)] comparison showed that response to abscisic acid and some stress-related biological processes such as regulation of response to stress, response to abiotic stimulus, regulation of defence response, and response to stress were enriched significantly (Figure 3B), whereas, the [S(−ABA)/C(−ABA)] comparison was enriched in different biological processes involved in response to abiotic stimulus, response to stress, response to oxidative stress, and response to abscisic acid (Figure 3C). Finally, response to hormones, the abscisic acid-activated signalling pathway, and response to endogenous stimulus were the most significant biological processes exclusively enriched in the [S(+ABA)/C(+ABA)] comparison (Figure 3D).

The abscisic acid-activated signalling pathway was exclusively enriched in the [S(+ABA)/C(+ABA)] comparison. A total of 14 DEGs were enriched in this pathway (Figure 3D). Seven of these 14 genes belonged to the kinase gene family including stress/ABA-activated protein kinases SAPK3 (LOC4349411, fold-change (FC) = 4.1), stress/ABA-activated protein kinases SAPK2 (LOC4343944, FC = −5.3), calcium-dependent calmodulin-independent protein kinase CDPK-like (LOC4339976, FC = −2.5), stress/ABA-activated protein kinases SAPK4 (LOC4324934, FC = 2.5), calcium-dependent protein kinase isoform 1 (LOC4349249, −2.7), calcium-dependent protein kinase, isoform 2 (LOC4349714, FC = 13.3), and serine/threonine-protein kinase SAPK7 (LOC4335877, FC = −6.1).

In addition, six genes that were enriched in the response to the abscisic acid biological process were also shared between [S(+ABA)/C(+ABA)], [S(+ABA)/S(−ABA)], and [S(−ABA)/C(−ABA)] comparisons. Four of these DEGs belonged to dehydrins, the including dehydrin family protein (LOC4350448), dehydrin Rab25 (LOC4326935), dehydrin Rab16C (LOC4350452), and dehydrin Rab16B (4350453). Two of those are members of the kinase gene family, namely calcium-dependent protein kinase isoform 1 (LOC4349249) and calcium-dependent protein kinase, isoform 2 (LOC4349714).

### 3.3. ABA-Altered Gene Regulation in Rice Plants

**Figure 4:**
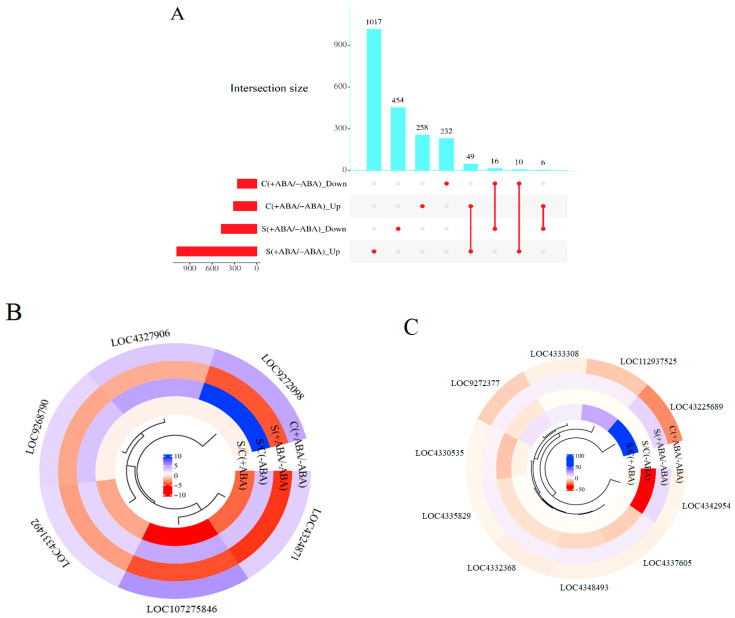
Differentially expressed genes (DEGs) in IAC1131 genotype in ABA-incubated plants relative to non-ABA-incubated plants (control/stress). (**A**) Upset diagram shows the number of genes up-regulated and down-regulated significantly in [C(+ABA)/C(−ABA)] and [S(+ABA)/S(−ABA)] comparisons. Histograms to the left of each sample set description correspond to the number of differentially expressed genes in each set. (**B**) Circular cluster heatmap of genes up-regulated in [C(+ABA)/C(−ABA)] and down-regulated in [S(+ABA)/S(−ABA)]. (**C**) Circular cluster heatmap of genes down-regulated in [C(+ABA)/C(−ABA)] and up-regulated in [S(+ABA)/S(−ABA)].

To decipher the genes responsible for ABA signalling in IAC1131 plants, a comparative study was performed between the ABA-incubated and non-incubated leaves, under both stress [S(+ABA)/S(−ABA)] and control [C(+ABA)/C(−ABA)] conditions (Figure 4). The ABA application induced a total of 2123 DEGs in comparison with normal conditions. About 25% (571) of these DEGs belonged to the control plants, [C(+ABA)/C(−ABA)], with 313 up-regulated and 258 down-regulated genes. Stress-treated plants incubated with ABA in comparison with non-ABA-incubated plants, [S(+ABA)/S(−ABA)], displayed approximately 2.5-fold more DEGs, consisting of 1076 up-regulated and 476 down-regulated genes (Table 2). The expression trends of the DEGs within individual comparisons between the control and stress-treated plants in response to ABA incubation followed similar trends, but at different levels in terms of the number of up-regulated and down-regulated genes. Interestingly, these results indicated that ABA-dependent DEGs showed a relatively similar expression pattern under stress and control circumstances. More importantly, the expression levels of the DEGs were obviously increased by stress in IAC1131 plants, suggesting that these DEGs could rapidly change their transcription in response to the switch from control conditions to multiple abiotic stress conditions, and hence might be involved in ABA signalling pathways (Figure 4A).

To identify the rice response to ABA incubation with respect to treatment conditions, we investigated the overlap in expression patterns between these two sets of comparisons. This process should find patterns of gene expression between stress and control treated plants, either with and without ABA incubation, including DEGs that were uniquely regulated in each comparison, similarly regulated in both comparisons, or oppositely regulated in both comparisons. Most DEGs were uniquely regulated under either control or stress treatment comparisons. Interestingly, out of 81 common DEGs, there were 16 DEGs regulated in an opposite manner in these two comparisons.

Six genes were up-regulated in the IAC1131 control ABA-incubated plants in comparison with the control non-ABA-incubated plants [C(+ABA)/C(−ABA)], but down-regulated in the stressed ABA-incubated plants in comparison with the stressed non-ABA-incubated plants [S(+ABA)/S(−ABA)]. These included rhomboid homologue (LOC4327906), lysine ketoglutarate reductase trans-splicing related 1 (LOC4324871), membrane associated DUF588 domain containing protein (LOC107275846), glycosylphosphatidylinositol anchor biosynthesis protein 11 (LOC4331492), and two unannotated expressed genes (LOC9268790 and LOC9272098) (Figure 4B). Taken together, these results may suggest that these genes play a role in helping IAC1131 plants tolerate unfavourable conditions, because their expression levels are correlated with both the imposition of stress and the application of a stress response hormone, when applied separately. However, they display opposite trends in expression between control and stress conditions after ABA incubation, suggesting that ABA pre-treatment does not necessarily induce them at higher expression levels in response to multiple abiotic stress.

Figure 4C provides detailed information regarding the 10 genes that overlapped between ABA-incubated and non-ABA-incubated plants under control and stress conditions. All of these genes were strongly up-regulated in response to ABA incubation in stressed plants [S(+ABA)/S(−ABA)] but down-regulated in the control plants when incubated with ABA [C(+ABA)/C(−ABA)]. This set of genes included serine/threonine-protein kinase receptor precursor (LOC4335829), 40S ribosomal protein S3-2 (LOC4333308), BTBN22 (LOC112937525), transposon protein (LOC4325684), cytochrome P450 (LOC4342954), LIM domain-containing protein (LOC4332368), ras-related protein (LOC4348493), choline monooxygenase (LOC9272377), and two unannotated expressed genes (LOC4337605 and LOC4330535). These genes responded positively to ABA application under stress conditions and are likely important in the core stress response through ABA signalling.

### 3.4. Stress-Regulated ABA-Induced Genes in Rice Plants

**Figure 5:**
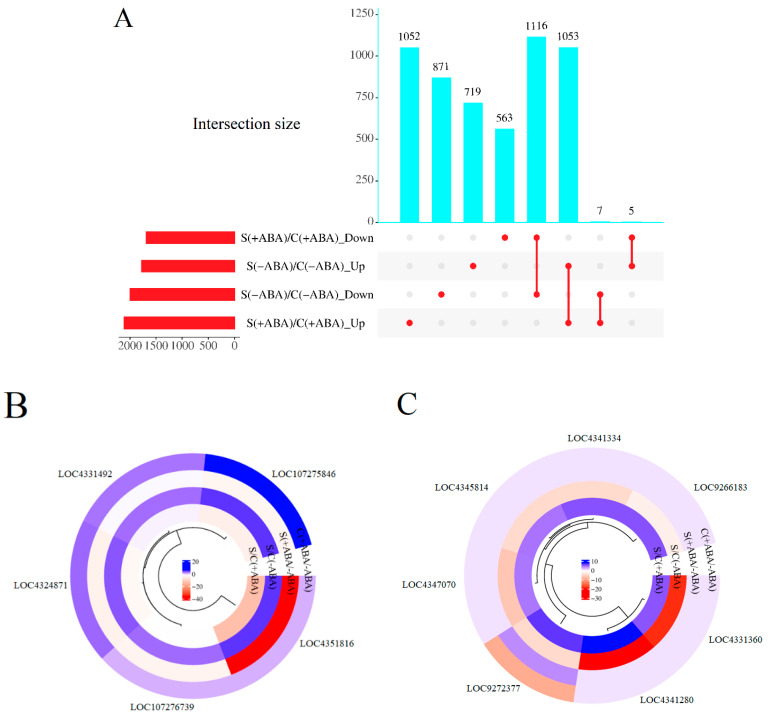
Differentially expressed genes (DEGs) in IAC1131 genotype in stressed plants relative to controls, with and without ABA incubation. (**A**) Upset diagram showing the number of genes up-regulated and down-regulated significantly in [S(−ABA)/C(−ABA)] and [S(+ABA)/C(+ABA)] comparisons. Histograms to the left of each sample set description correspond to the number of differentially expressed genes in each set. (**B**) Circular cluster heatmap of genes up-regulated in [S(−ABA)/C(−ABA)] and down-regulated in [S(+ABA)/C(+ABA)]. (**C**) Circular cluster heatmap of genes down-regulated in [S(−ABA)/C(−ABA)] and up-regulated in [S(+ABA)/C(+ABA)].

To address the impact of prior exposure to exogenous ABA on the rice transcriptome when exposed to multiple abiotic stressed, we analysed gene expression in ABA-inoculated plants grown in normal and multiple abiotic stress conditions, compared with non-ABA-inoculated plants grown in normal and multiple abiotic stress conditions. The comparison between the stressed and control IAC1131 plants in the absence of ABA pre-treatment, [S(−ABA)/C(−ABA)], revealed that stress by itself greatly altered the transcriptomic profile, involving more down-regulation of genes. Conversely, the similar comparison, but with ABA incubation [S(+ABA)/C(+ABA)], revealed significant alterations in the expression of DEGs, a majority of which were up-regulated (Figure 5A).

Differential expression analysis revealed more than 3700 DEGs in each of these two comparisons (Table 2). A majority of DEGs, 2112 genes, were up-regulated in the [S(+ABA)/C(+ABA)] comparison, while a majority of DEGs, 1994 genes, were down-regulated in the [S(−ABA)/C(−ABA)] comparison (Table 2, Figure 5A). This suggests that plants responded in a complex fashion to combined abiotic stress by regulating expression of many genes. This included both up-regulation and down-regulation, but ABA pre-treatment may be assisting in stress response by switching the expression of a number of genes from down-regulation to up-regulation.

Within each comparison of stressed plants compared to controls, there were shared and unique transcriptomic responses in both the ABA-incubated and non-ABA-incubated groups (Figure 5A). There were a great number of unique DEGs specific to individual comparisons (1052 + 871 + 719 + 563 = 3205), and a large number of DEGs that overlapped between the two comparisons (1116 + 1053 + 7 + 5 = 2181). This finding signified that multiple abiotic stress was the dominant factor affecting IAC1131 transcriptome response. Moreover, while exposure to multiple abiotic stress caused significant changes in the transcriptome profile and gene expression in rice plants, ABA application mitigated the impact of stress on plants by up-regulation of specific stress-responsive genes. Notably, almost all the DEGs shared between both comparisons (2169 genes, 99.5%), [S(+ABA)/C(+ABA)], and [S(−ABA)/C(−ABA)], were either up-regulated in both or down-regulated in both. There were only 12 genes (0.5%) that were altered in expression in opposite directions between the two comparisons.

Five of these were up-regulated in response to stress in the absence of ABA, but down-regulated in response to stress with ABA pre-treatment (Figure 5B). This included glycosylphosphatidylinositol anchor biosynthesis protein 11 (LOC4331492), membrane-associated protein (LOC107275846), tetratricopeptide repeat-containing protein (LOC107276739), NADP-dependent oxidoreductase (LOC4351816), and lysine ketoglutarate reductase trans-splicing related 1 (LOC4324871). Conversely, seven genes responded positively to ABA incubation and exposure to multiple abiotic stress and were up-regulated under [S(+ABA)/C(+ABA)] comparison, but down-regulated in the absence of ABA pre-treatment (Figure 5C). These included folylpolyglutamate synthase (LOC4331360), SPOC domain-containing protein (LOC4341334), kinesin motor domain-containing protein (LOC4341280), choline monooxygenase (LOC9272377), retrotransposon protein (LOC9266183), WRKY62 (LOC4347070), and alpha-amylase precursor (LOC4345814). The contrasting expression pattern of these 12 genes shows that ABA signalling is likely important in stress response in rice plants, and interaction between the ABA signalling pathway and stress-responsive genes can be reflected in the plant transcriptome profile.

### 3.5. Connections between ABA-Related Genes and Pathways and Stress-Responsive Genes

**Figure 6:**
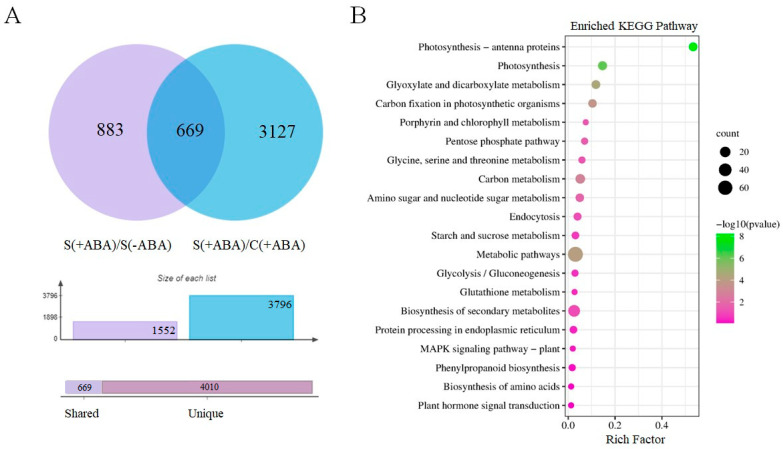
Differential gene expression analysis between [S(+ABA)/S(−ABA)] and [S(+ABA)/C(+ABA)] comparisons (SA). (**A**) Venn diagram of DEGs. (**B**) Top 20 KEGG pathways of the 669 common differentially expressed genes. The size of the dot indicates the number of DEGs involved in the pathway. The colour scale indicates the significance level based on corrected -log10 *p*-value. The *y*-axis indicates the pathway name, the *x*-axis indicates the enrichment factor in each of the pathways, and the bubble size indicates the number of genes.

Since phytohormones like ABA are known regulators of plant responses, we investigated how stress responses were influenced by phytohormone-induced genes. This involved examining the commonalities between genes differentially expressed between stressful conditions with and without ABA pre-treatment, and those genes altered in response to stress relative to control, with ABA pre-treatment. In total, there were 669 DEGs shared between the [S(+ABA)/S(−ABA)] and [S(+ABA)/C(+ABA)] comparisons, which can be considered as genes expressed as part of the interaction between multiple abiotic stress and ABA accumulation (Figure 6A). KEGG enrichment analysis for the DEGs in common between these two comparisons were assigned into 80 KEGG pathways. The top 20 enriched KEGG pathways terms in common between [S(+ABA)/S(−ABA)] and [S(+ABA)/C(+ABA)] are shown in Figure 6B. Photosynthesis and photosynthesis antenna protein-related genes, along with genes related to the biosynthesis of secondary metabolites, were significantly enriched in common between [S(+ABA)/S(−ABA)] and [S(+ABA)/C(+ABA)], an intersecting set referred to hereafter as SA.

**Figure 7:**
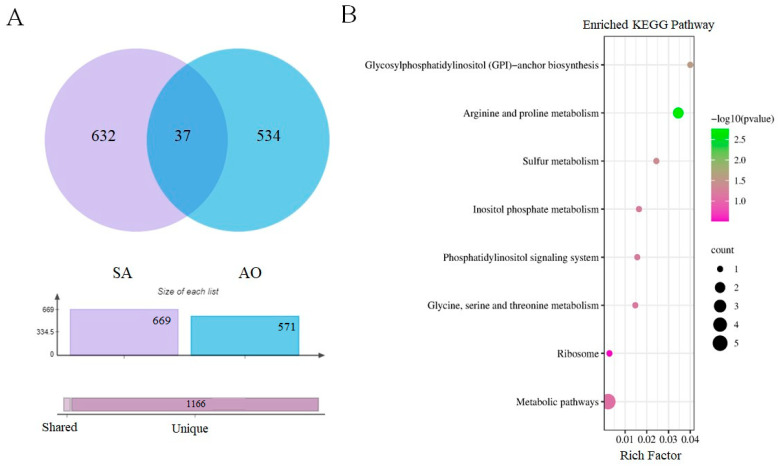
Differential gene expression analysis between SA and AO comparisons. (**A**) Venn diagram of DEGs. (**B**) KEGG pathways of the 37 common differentially expressed genes. The size of the dot indicates the number of DEGs involved in the pathway. The colour scale indicates the significance level based on the corrected -log10 *p*-value. The *y*-axis indicates the pathway name, the *x*-axis indicates the enrichment factor in each of the pathways, and the bubble size indicates the number of genes.

Comparing ABA-incubated and non-ABA-incubated rice plants under control conditions [C(+ABA)/C(−ABA)], a comparison set referred to hereafter as AO, resulted in a total of 571 DEGs, which can be considered as ABA-dependent genes. Comparisons between DEGs of SA and AO resulted in only 37 common DEGs, which are likely to be genes in the SA set that are related to ABA signalling (Figure 7A). KEGG pathway analysis was performed for these common DEGs and a total of eight significant KEGG pathways were enriched for the 37 DEGs (Figure 7B) including glycosylphosphatidylinositol (GPI)-anchor biosynthesis, arginine and proline metabolism, sulphur metabolism, inositol phosphate metabolism, the phosphatidylinositol signalling system, glycine, serine, and threonine metabolism, and ribosome. Notably, the 669 DEGs from SA and 37 DEGs common between SA and AO were enriched in completely different GO terms, except for the glycine, serine, and threonine metabolism pathway. Only one gene related to the latter pathway was present in the 37 DEGs in common between SA and AO, but four genes related to this pathway were enriched from 669 DEGs in the SA comparison.

**Figure 8:**
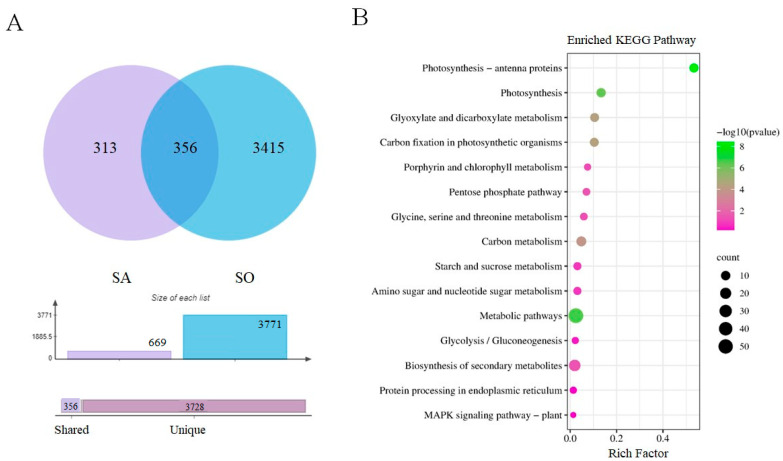
Differential gene expression analysis between SA and SO comparisons. (**A**) Venn diagram of DEGs. (**B**) Top 15 KEGG pathways of the 356 common differentially expressed genes. The size of the dot indicates the number of DEGs involved in the pathway. The colour scale indicates the significance level based on the corrected −log10 *p*-value. The *y*-axis indicates the pathway name, the *x*-axis indicates the enrichment factor in each of the pathways, and the bubble size indicates the number of genes.

To assess the plant transcriptome response to stress and interaction with ABA signalling pathways, shared expressed genes were extracted from the DEGs of [S(−ABA)/C(−ABA)] (referred to hereafter as SO) and SA, a comparison which involved 3771 and 669 DEGs, respectively. There were 356 genes discovered in common between the SA and SO comparisons (Figure 8A). Similar to previous comparisons, we evaluated the enrichment patterns of the DEGs shared between the SA and SO comparisons in terms of responsive KEGG pathways. The results revealed 58 enriched KEGG pathways, the top 15 of which are presented in Figure 8B.

Although some KEGG pathways were enriched exclusively in the SA comparison, all of the top 15 enriched pathways in the SO comparison were also identified in the SA comparison, including metabolic pathways, biosynthesis of secondary metabolites, carbon metabolism, photosynthesis, photosynthesis—antenna proteins, glyoxylate and dicarboxylate metabolism, carbon fixation in photosynthetic organisms, starch and sucrose metabolism, amino sugar and nucleotide sugar, pentose phosphate, glycine, serine and threonine metabolism, porphyrin and chlorophyll metabolism, glycolysis/gluconeogenesis, protein processing in endoplasmic reticulum, and MAPK signalling.

These results indicated that stress-responsive genes were involved in many aspects of plant responses such as photosynthesis, secondary metabolism, carbon metabolism, carbohydrate metabolism, and signalling. DEGs related to the top KEGG enriched pathways common between the different comparisons may play important roles in plant response to multiple abiotic stress. Moreover, multiple abiotic stress was the dominant factor altering the plant transcriptome, however, ABA incubation can also mitigate the effects of environmental stress by enriching unique pathways and forming regulatory networks in cooperation with stress-responsive genes.

### 3.6. Functional Classification of Common DEGs between Stress and ABA Signalling

To investigate the rice biological processes altered during stress, we evaluated the enrichment patterns of genes differentially expressed under SA comparison (669), common between SA and SO (356), and common between SA and AO (37). Gene ontology (GO) functional classification was performed to determine the biological processes that the common DEGs were involved in, which identified six, 19 and zero GO biological process terms, respectively, and biological processes that were significantly enriched among common DEGs in the SA, (SA and SO), and (SA and AO) data sets.

**Figure 9:**
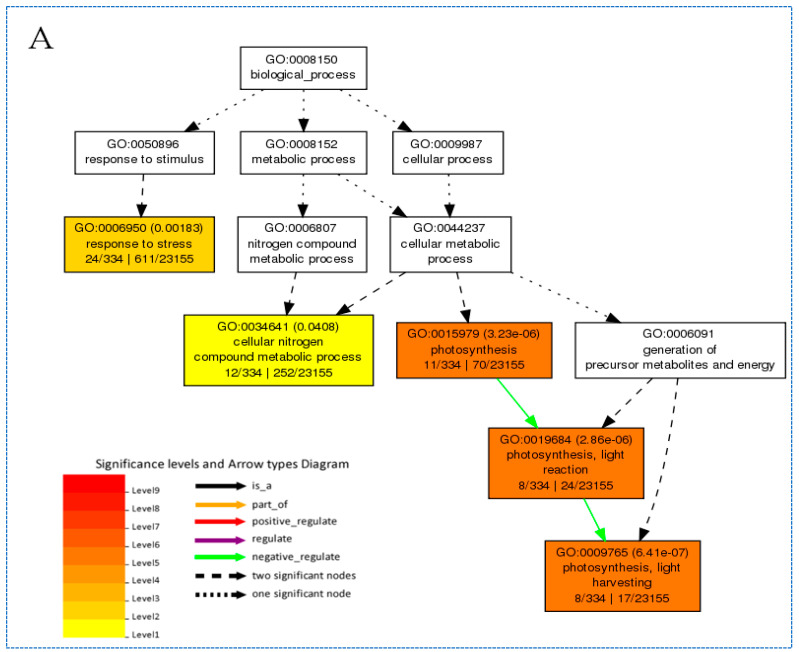

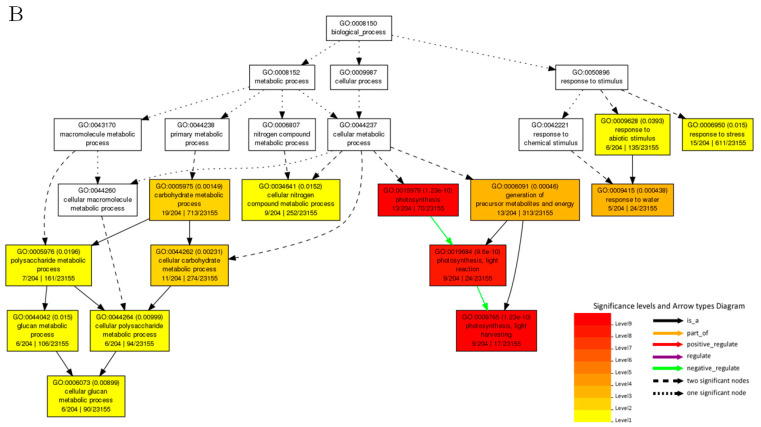
GO enrichment analysis for DEGs. (**A**) The categories of biological processes GO terms enriched in common under SA comparison; the darker the box colour, the higher the significance level. (**B**) The categories of biological process GO terms enriched in common in (SA and SO); the darker the box colour, the higher the significance level.

The GO terms ‘photosynthesis’, ‘photosynthesis, light harvesting’, ‘photosynthesis, light reaction’, ‘response to stress’, ‘chloroplast’, and ‘cellular nitrogen compound metabolic process’ were all enriched within DEGs by both SA and (SA and SO) comparisons (Figure 9), but no GO terms were enriched in genes differentially regulated in the (SA and AO) comparisons. Altered regulation of photosynthetic pathways is a common rice response to stress and these results show that ABA accumulation can positively regulate related genes. Importantly, GO functional analysis indicated that photosynthetic activity was decreased under stress treatment and also in response to ABA application. Another finding of enrichment analysis was related to the different levels of enrichment between the SA and (SA and SO) comparisons. The significance level of enriched GO categories related to photosynthesis, photosynthesis (light reaction), and photosynthesis (light harvesting) from SA was lower in common genes also regulated in (SA and SO). Consequently, ABA efficacy in affording plant tolerance against multiple abiotic stress can be seen in the number of DEGs, the number of up-regulated genes, and the enrichment of related biological processes in plants.

## 4. Discussion

A variety of studies have sounded a note of caution about the serious loss of crop yield attributable to environmental stresses [2,33,34], and exogenous application of ABA can enhance the abiotic stress tolerance of plants [35]. With the increase in extreme weather events due to climate change and the constant pressure of urbanization, there is an urgent need to develop crop varieties that are more tolerant to environmental stresses. Dynamic transcriptome expression represents the degree of gene expression in plants at each growing stage, or each specific environmental condition. Transcriptome analysis can clarify the intricate regulatory networks relating to the stress tolerance and adaptability of plants, including those related to ABA metabolism [36].

A variety of environmental stresses affect plants in the field and can limit crop yield. To endure these stresses, plants respond with coordinated changes to their transcriptome. In this study, to unveil the impact of ABA pre-treatment on molecular regulation in rice plants exposed to multiple abiotic stress, we conducted a quantitative transcriptome analysis to identify modifications in gene expression in response to simultaneous abiotic stresses following exogenous ABA application on leaves of the rice IAC1131 genotype.

To characterize the effect of ABA application on plant molecular mechanisms under control (non-stressed) conditions, we compared ABA-incubated and non-ABA-incubated plants (Figure 4A). ABA is an important phytohormone regulating plant growth, development, and stress responses through multiple signalling pathways [37], and a basal ABA level is essential in the regulation of plant growth at different levels in various tissues [38]. An increase in endogenous ABA accompanies adaptative responses to environmental stresses, and exogenously applied ABA can assist plants in responding to, and surviving under, environmental stress [39]. Our results revealed that ABA overaccumulation in the IAC1131 control plants did not alter the transcriptome profile greatly, however, there were a higher number of up-regulated genes than down-regulated genes.

Exogenous application of ABA on stressed plants induced a very large number of changes in gene expression, with more genes up-regulated than down-regulated in plants incubated with ABA when compared to non-ABA-incubated plants (Figure 4A). In agreement with this, a previous study showed that ABA positively affects stress tolerance following exogenous application, or through the overexpression of genes for increased endogenous ABA content in plants [40]. A rapid increase of ABA under stressful conditions triggers a series of physiological responses and signalling transduction in plants [41]. Essentially, an increase in ABA biosynthesis in plants is a sign of an environmental imbalance like abiotic stress, which plays a role in inhibiting ABA degradation and is thought to be stimulated by stress relief.

Although ABA application increased the number of up-regulated genes to a greater level than down-regulated genes in both the control and stressed plants, the number of DEGs differed remarkably between plants grown in control or stress conditions. ABA incubation in stressed plants resulted in an increase in total DEGs to more than 2.7 times in comparison with the control samples, while the number of exclusively up-regulated genes increased to about 3.5 times. This result shows that while an increase in ABA level might not have a significant effect on plants in non-stress conditions, application of exogenous ABA certainly appears to be important in acclimation to stress. Therefore, manipulating ABA levels, along with the associated signalling pathways, has the potential to generate useful crop varieties with improved productivity in undesirable environments [42].

An opposite trend in gene expression between stressed and control plants incubated with ABA, in comparison with non-ABA-incubated plants, illustrated new findings about genes responsive to stress and ABA signalling. For example, the transposon protein gene (LOC4325684) responded positively to ABA incubation in stressed plants, unlike in control plants incubated with ABA in comparison with untreated plants (Figure 4C). Transposable elements (TE) are responsible for very important roles in genome evolution in many plant species. Several TEs are known as gene regulators by influencing the expression of genes as stress-responsive regulatory motifs [43]. Another recent finding illustrated that the mobilization of stress-induced transposable elements is potentially important as they may play a role in stress adaptation, by taking part in novel gene regulatory pathways responding to various stresses [44].

Transposon genes are also predicted to be more expressed in response to stress. In our experiment, however, we observed down-regulation of a transposon gene to a very low level in control plants incubated with ABA, in comparison with non-ABA-incubated control samples. This warrants further investigation, to determine if there is any correlation between ABA overaccumulation in untreated plants and the expression of TE factors.

The BTBN22 gene (Broad Complex BTB domain with non-phototropic hypocotyl 3 NPH3 and coiled-coil domains) (LOC112937525) (BTB) showed contrasting expression patterns in plants grown in stressed and control conditions. ABA application on stressed plants induced increased expression of this gene, while a decline in gene expression was seen in ABA-treated plants grown in control conditions (Figure 4C). One of the main functions of BTB is involvement in many biological processes in plants coordinated with abiotic stress response [45]. Furthermore, the BTB domain protein-related gene negatively regulates ABA-mediated inhibition in plants, possibly by repressing the expression of a subset of ABA-dependent genes [46]. Interestingly, both ABA and BTB were identified as stress-responsive elements, but ABA overaccumulation in control conditions could lead to negative regulation of the BTB gene.

Six genes showed contrasting expression patterns between the control and stress comparison of ABA-incubated and non-ABA-incubated IAC1131 plants. Of these, the membrane-associated DUF588 domain-containing protein gene (LOC107275846), was up-regulated the most in control plants, and reduced significantly in the stress plant comparison (Figure 4B). The DUF588 domain-containing protein gene belongs to the DUF family of genes, which plays a possible role in plant leaf development. Studies have shown that the main gene responsible for leaf rolling *(REL1)* might coordinate with the ABA pathway to regulate drought tolerance in rice plants. Deletion of the *REL1* down-regulated a membrane-associated DUF588 domain-containing protein, and a closely related gene, *REL2*, encodes for a protein containing DUF630 and DUF632 domains. [47]. Interestingly, some REL1 regulators are ABA-independent, but still modulate drought tolerance in plants [48]. Our findings showed that ABA application on control plants correlated positively with the expression of this DUF588 domain-containing protein gene, while exogenous ABA did not lead to increased expression of this gene in stressed plants. Therefore, both ABA application and stress conditions might be expected to induce expression of the DUF588 domain-containing protein gene.

Comparative transcriptome analysis revealed many more DEGs during multiple abiotic stress in comparison with control plants, whether ABA incubated or non-ABA incubated (Figure 5A). Although DEGs under [S(−ABA)/C(−ABA)] and [S(+ABA)/C(+ABA)] were similar in terms of numbers, ABA application resulted in fewer down-regulated genes than up-regulated genes. This shows the positive role of ABA in balancing the negative impact of combined abiotic stress on IAC1131 plants.

A total of 12 genes were commonly differentially expressed, but in contrasting directions, in ABA-incubated and non-ABA-incubated comparisons of stressed control IAC1131 plants relative to control plants. Five of these 12 were up-regulated in response to stress without prior ABA application, but down-regulated to a low level in response to stress after ABA application (Figure 5B). For example, NADP-dependent oxidoreductase (LOC4351816) was up-regulated (FC = 4.2) without ABA pre-treatment, and down-regulated (FC = −14.4) after ABA application. This result illustrates that while multiple abiotic stress induced the activation of NADP-dependent oxidoreductase, ABA pre-treatment suppressed gene expression.

Reactive oxygen species (ROS) are known as one of the most important players in plant response to stresses. ROS function as second messengers to positively regulate ABA signalling, while on the other hand, some NADP-dependent oxidoreductases are responsible for ABA-induced ROS production [49]. A previous study in Arabidopsis identified a mutation in the gene for quinolinate synthase, which is critical in NAD biosynthesis, that caused hypersensitivity to salt stress and ABA. The ABA hypersensitivity could be rescued by supplementation of the NAD precursor, while ABA induced overaccumulation of ROS in the mutant plants. This reflects the tightly integrated regulatory network connecting NAD biosynthesis, ABA signalling, and ROS production [50]. Interestingly, our results confirmed the positive role of the NADP-dependent oxidoreductase gene in stress response in the IAC1131 plants, but ABA incubation suppressed its activity, even under stress conditions.

Conversely, kinesin motor domain-containing protein gene (LOC4341280) was up-regulated (FC = 7.5) in a comparison of stressed plants relative to control plants incubated with ABA, but substantially down-regulated (FC = −20.4) in the absence of ABA application (Figure 5C). Kinesins are a conserved superfamily of microtubule-dependent motor proteins in most eukaryotes. They contribute to diverse essential functions in plant cells, including cell development and intracellular transport [51]. Although few studies have focused on the regulation of kinesins in response to stress, a kinesin light chain-related gene has been reported to induce sensitivity to drought stress in Arabidopsis [52]. Notably, no previous reports have mentioned any correlation between kinesin genes and phytohormone signalling in plants. Our results confirmed the negative affect of multiple abiotic stress on a kinesin motor domain-containing protein gene, but ABA overaccumulation induces this gene even with stress treatment. This may reflect a regulatory mechanism involving the ABA signalling pathway and kinesin genes in rice plants, which makes this gene an interesting candidate for more detailed future mechanistic studies.

Analysing genes that are commonly altered in expression under different conditions provides a way to identify genes that are responsive to ABA signalling and multiple abiotic stress simultaneously. It also allows for the identification of ABA-dependent and independent signalling pathways, which play active roles in stress response in plants. ABA signalling pathways are activated immediately when plants sense abiotic stress, and interact with stress responsive cascades via the induction of ABA-responsive genes. ABA signalling pathways integrate with other important signalling pathways, like those related to environmental stress responses in plants. Our results showed that the expression of SAPK genes was related to the ABA signalling pathway biological process, however the expression level of SAPK2, SAPK3, SAPK4, and SAPK7 genes was altered exclusively in the comparison between stress and control IAC1131 plants incubated with ABA. This finding suggests that the expression of SAPK genes in response to multiple abiotic stress is mediated by ABA. Previous studies have reported that SAPKs are identified as one the four core components of ABA-dependent gene expression signalling in plants [53]. For instance, overexpression of the SAPK2 gene is correlated with abiotic stress tolerance through the promotion of stomatal closure and modification in some of the dehydrin genes [54].

Rab16b and OsRab21, drought resistance-related genes encoding for dehydrin family members, also play important roles in ABA-dependent stress tolerance in plants [55,56,57]. Interestingly, four proteins belonging to the gene family expressed significantly in response to abiotic stress treatment both with and without ABA incubation. A recent study revealed that dehydrin genes can be categorized as either ABA-dependent or ABA-independent genes [56].

The Ca^2+^ dependent protein kinases (CDPKs) are key components of stomatal closure regulation. Stomatal closure is known as an ABA-mediated abiotic stress response [58,59]. The altered expression level of calcium-dependent protein kinase, isoform 2, and calcium-dependent protein kinase, isoform 1, genes showed that CDPKs are responsive to ABA in rice plants. However, significant expression of the same genes in [S(−ABA)/C(−ABA)] indicates that a basal level of ABA may have an effect on CDPK gene function, resulting in overexpression in response to environmental stress. Linking all of these together, it has been demonstrated that dehydrin gene expression under stress conditions is dependent on interaction between the ABA signalling pathways, kinase gene cascades, and the Ca^2+^ signalling pathways [60].

DEGs in the SA comparison (significantly enriched in common between [S(+ABA)/S(−ABA)] and [S(+ABA)/C(+ABA)]), were enriched in similar GO functions to those differentially expressed in the SO comparison, [S(−ABA)/C(−ABA)]. This indicates that multiple abiotic stress appears to be the dominant driver of plant transcriptome alteration, since DEGs responsive to ABA overaccumulation were similar to stress-responsive genes. In this case, ABA could be considered as one of the stress response elements.

Both KEGG pathway analysis and GO functional analysis revealed photosynthesis-related mechanisms as a major biological function interacting with both ABA signalling and stress response in plants. This is in good agreement with previous studies, as exogenous ABA application has been shown to influence photosynthetic parameters and photosynthetic regulation in pea seedlings, while ABA reduced the photosynthetic capacity of plants by modulating RuBisCO activity and promoting stomatal closure [61]. Based on this, it appears likely that ABA might be an important communication link between environmental stress response and reductions in photosynthetic capacity in plants [62,63].

Taken together, these results showed that a basal level of ABA is essential for stress-escape mechanisms in plants under non-stressful conditions, but an increase in amount of ABA in plants triggers a stress response, which can result in a reduction in photosynthesis and gas exchange activities. This finding is congruent with an earlier study that found an increase of ABA level in response to unfavourable conditions changes the plant profile from growth to survival by energy conservation through various mechanisms, including photosynthesis function reduction [64]. Therefore, ABA is not only a stress-responsive hormone but also plays an important role in non-stressful conditions in rice plants.

## 5. Conclusions

In summary, this study provides a comprehensive overview of the transcriptome of the IAC1131 rice genotype in response to multiple abiotic stress, with and without prior ABA incubation, and highlights the transcriptional variations that occur. ABA is an important hormone in response to stress in plants, and the results revealed that the ABA pathway was activated during multiple abiotic stress through a synergistic interaction with stress response in plants. The addition of exogenous ABA under non-stressed conditions did not substantially change the transcriptome, while combined abiotic stress caused more damage to non-ABA-incubated control plants than ABA-incubated plants. The down-regulation of physiological pathways such as photosynthesis illustrated that ABA mediated the mitigation of the effects of combined abiotic stress on rice plants, suggesting that enhanced stress resistance is partially dependent on ABA signalling.

The extensive transcriptome divergence, including differential gene expression patterns, GO functional analysis, and KEGG metabolic pathways, suggests ABA pre-treatment may have positive effects which could help to induce tolerance against environmental stresses. We identified some common and exclusive molecular functions in the stress responsive and ABA signalling pathways, which may be important for sensitivity or tolerance to parallel abiotic stress in rice. The data generated in this study have highlighted numerous candidate biomarker genes that are related to both ABA signalling and stress-responsive metabolisms. These genes can be validated through multi-omics analysis and investigated in future gene editing and marker-assisted selective breeding programs, with the aim of enhancing stress tolerance in rice.

## Figures and Tables

**Table 1:** Summary of read data, mapping, and reference-based assembly obtained for IAC1131 samples (Rep = biological replicate).

Condition	Raw Reads			Trimmed Reads (%)			Uniquely Mapped Reads (%)		
	Rep1	Rep2	Rep3	Rep1	Rep2	Rep3	Rep1	Rep2	Rep3
(Control − ABA)	28,681,613	24,843,962	33,914,978	99.02	99.03	99.10	94.79	92.43	91.51
(Control + ABA)	35,537,575	32,563,746	42,836,163	99.15	99.07	99.15	92.77	93.45	95.19
(Stress − ABA)	19,788,477	16,515,788	32,333,428	99.02	99.04	99.00	73.73	83.73	89.74
(Stress + ABA)	32,496,847	34,908,908	39,286,881	99.03	99.07	99.16	90.65	89.86	91.22

**Table 2:** Genes differentially expressed in IAC1131 rice plants in four different comparisons.

Stress Treatments Comparison	DEGs	Up-Regulated	Down-Regulated
[C(+ABA)/C(−ABA)]	571	313	258
[S(+ABA)/S(−ABA)]	1552	1076	476
[S(−ABA)/C(−ABA)]	3771	1777	1994
[S(+ABA)/C(+ABA)]	3796	2112	1684

## Data Availability

RNA-seq data have been deposited to the National Centre for Biotechnology Information via the NCBI submission portal with BioProject identifier SUB12326911.

## References

[1] Nawaz G., Kang H. (2019). Rice OsRH58, a chloroplast dead-box RNA helicase, improves salt or drought stress tolerance in arabidopsis by affecting chloroplast translation. BMC Plant Biol..

[2] Cohen S.P., Leach J.E. (2019). Abiotic and biotic stresses induce a core transcriptome response in rice. Sci. Rep..

[3] Habibpourmehraban F., Wu Y., Wu J.X., Hamzelou S., Masoomi-Aladizgeh F., Kamath K.S., Amirkhani A., Atwell B.J., Haynes P.A. (2022). Multiple abiotic stresses applied simultaneously elicit distinct responses in two contrasting rice cultivars. Int. J. Mol. Sci..

[4] Dar M.H., Bano D.A., Waza S.A., Zaidi N.W., Majid A., Shikari A.B., Ahangar M.A., Hossain M., Kumar A., Singh U.S. (2021). Abiotic stress tolerance-progress and pathways of sustainable rice production. Sustainability.

[5] Dolferus R. (2014). To grow or not to grow: A stressful decision for plants. Plant Sci..

[6] Park J., Oh S.-K., Chung H.J., Park H.-J. (2020). Structural and physicochemical properties of native starches and non-digestible starch residues from korean rice cultivars with different amylose contents. Food Hydrocoll..

[7] Mareri L., Parrotta L., Cai G. (2022). Environmental stress and plants. Int. J. Mol. Sci..

[8] Zhou R., Wan H., Jiang F., Li X., Yu X., Rosenqvist E., Ottosen C.O. (2020). The alleviation of photosynthetic damage in tomato under drought and cold stress by high CO_2_ and melatonin. Int. J. Mol. Sci..

[9] Wang Y., Wang S., Tian Y., Wang Q., Chen S., Li H., Ma C., Li H. (2021). Functional characterization of a sugar beet bvbhlh93 transcription factor in salt stress tolerance. Int. J. Mol. Sci..

[10] Wang X., Zhang M., Xie B., Jiang X., Gai Y. (2021). Functional characteristics analysis of dehydrins in *Larix kaempferi* under osmotic stress. Int. J. Mol. Sci..

[11] Wang H.L., Chekanova J.A. (2016). Small RNAs: Essential regulators of gene expression and defenses against environmental stresses in plants. Wiley Interdiscip. Rev. RNA.

[12] Xie Z., Nolan T.M., Jiang H., Yin Y. (2019). Ap2/erf transcription factor regulatory networks in hormone and abiotic stress responses in arabidopsis. Front. Plant Sci..

[13] Virlouvet L., Avenson T.J., Du Q., Zhang C., Liu N., Fromm M., Avramova Z., Russo S.E. (2018). Dehydration stress memory: Gene networks linked to physiological responses during repeated stresses of *Zea mays*. Front. Plant Sci..

[14] Mitsuda N., Ohme-Takagi M. (2009). Functional analysis of transcription factors in arabidopsis. Plant Cell Physiol..

[15] Li R., Fu D., Zhu B., Luo Y., Zhu H. (2018). Crispr/cas9-mediated mutagenesis of lncRNA1459 alters tomato fruit ripening. Plant J..

[16] Wu Y., Mirzaei M., Pascovici D., Chick J.M., Atwell B.J., Haynes P.A. (2016). Quantitative proteomic analysis of two different rice varieties reveals that drought tolerance is correlated with reduced abundance of photosynthetic machinery and increased abundance of CLPd1 protease. J. Proteom..

[17] Wu Y., Mirzaei M., Pascovici D., Haynes P.A., Atwell B.J. (2019). Proteomes of leaf-growing zones in rice genotypes with contrasting drought tolerance. Proteomics.

[18] Han X., Wu Z., Liu F., Wang Y., Wei X., Tian P., Ling F. (2023). Transcriptomic analysis and salt-tolerance gene mining during rice germination. Genes.

[19] Dwivedi A.K., Singh V., Anwar K., Pareek A., Jain M. (2023). Integrated transcriptome, proteome and metabolome analyses revealed secondary metabolites and auxiliary carbohydrate metabolism augmenting drought tolerance in rice. Plant Physiol. Biochem..

[20] Wang J., Hu K., Wang J., Gong Z., Li S., Deng X., Li Y. (2023). Integrated transcriptomic and metabolomic analyses uncover the differential mechanism in saline-alkaline tolerance between indica and japonica rice at the seedling stage. Int. J. Mol. Sci..

[21] Leggett R.M., Ramirez-Gonzalez R.H., Clavijo B.J., Waite D., Davey R.P. (2013). Sequencing quality assessment tools to enable data-driven informatics for high throughput genomics. Front. Genet..

[22] Bolger A.M., Lohse M., Usadel B. (2014). Trimmomatic: A flexible trimmer for illumina sequence data. Bioinformatics.

[23] Grabherr M.G., Haas B.J., Yassour M., Levin J.Z., Thompson D.A., Amit I., Adiconis X., Fan L., Raychowdhury R., Zeng Q. (2011). Full-length transcriptome assembly from rna-seq data without a reference genome. Nat. Biotechnol..

[24] Kriventseva E.V., Kuznetsov D., Tegenfeldt F., Manni M., Dias R., Simao F.A., Zdobnov E.M. (2019). Orthodb v10: Sampling the diversity of animal, plant, fungal, protist, bacterial and viral genomes for evolutionary and functional annotations of orthologs. Nucleic Acids Res..

[25] Seppey M., Manni M., Zdobnov E.M. (2019). Busco: Assessing genome assembly and annotation completeness. Methods Mol. Biol..

[26] Haas B.J., Papanicolaou A., Yassour M., Grabherr M., Blood P.D., Bowden J., Couger M.B., Eccles D., Li B., Lieber M. (2013). *De novo* transcript sequence reconstruction from RNA-seq using the trinity platform for reference generation and analysis. Nat. Protoc..

[27] Dobin A., Davis C.A., Schlesinger F., Drenkow J., Zaleski C., Jha S., Batut P., Chaisson M., Gingeras T.R. (2013). Star: Ultrafast universal RNA-seq aligner. Bioinformatics.

[28] Li B., Dewey C.N. (2011). Rsem: Accurate transcript quantification from RNA-seq data with or without a reference genome. BMC Bioinform..

[29] Langmead B., Salzberg S.L. (2012). Fast gapped-read alignment with bowtie 2. Nat. Methods.

[30] Tian T., Liu Y., Yan H., You Q., Yi X., Du Z., Xu W., Su Z. (2017). Agrigo v2.0: A go analysis toolkit for the agricultural community, 2017 update. Nucleic Acids Res..

[31] Robinson M.D., McCarthy D.J., Smyth G.K. (2010). Edger: A bioconductor package for differential expression analysis of digital gene expression data. Bioinformatics.

[32] Bu D., Luo H., Huo P., Wang Z., Zhang S., He Z., Wu Y., Zhao L., Liu J., Guo J. (2021). Kobas-i: Intelligent prioritization and exploratory visualization of biological functions for gene enrichment analysis. Nucleic Acids Res..

[33] Li N., Zhang Z., Chen Z., Cao B., Xu K. (2021). Comparative transcriptome analysis of two contrasting chinese cabbage (*Brassica rapa L*.) genotypes reveals that ion homeostasis is a crucial biological pathway involved in the rapid adaptive response to salt stress. Front. Plant Sci..

[34] Liang Y., Tabien R.E., Tarpley L., Mohammed A.R., Septiningsih E.M. (2021). Transcriptome profiling of two rice genotypes under mild field drought stress during grain-filling stage. AoB Plants.

[35] Kanehisa M., Goto S., Kawashima S., Nakaya A. (2002). The KEGG databases at genomenet. Nucleic Acids Res..

[36] Ahmad M. (2022). Genomics and transcriptomics to protect rice (*Oryza sativa.* L.) from abiotic stressors: -pathways to achieving zero hunger. Front. Plant Sci..

[37] Chen K., Li G.J., Bressan R.A., Song C.P., Zhu J.K., Zhao Y. (2020). Abscisic acid dynamics, signaling, and functions in plants. J. Integr. Plant Biol..

[38] Brookbank B.P., Patel J., Gazzarrini S., Nambara E. (2021). Role of basal aba in plant growth and development. Genes.

[39] Chen C.W., Yang Y.W., Lur H.S., Tsai Y.G., Chang M.C. (2006). A novel function of abscisic acid in the regulation of rice (*Oryza sativa* L.) root growth and development. Plant Cell Physiol..

[40] Vishwakarma K., Upadhyay N., Kumar N., Yadav G., Singh J., Mishra R.K., Kumar V., Verma R., Upadhyay R.G., Pandey M. (2017). Abscisic acid signaling and abiotic stress tolerance in plants: A review on current knowledge and future prospects. Front. Plant Sci..

[41] Li P., Yang H., Wang L., Liu H., Huo H., Zhang C., Liu A., Zhu A., Hu J., Lin Y. (2019). Physiological and transcriptome analyses reveal short-term responses and formation of memory under drought stress in rice. Front. Genet..

[42] Miao C., Xiao L., Hua K., Zou C., Zhao Y., Bressan R.A., Zhu J.K. (2018). Mutations in a subfamily of abscisic acid receptor genes promote rice growth and productivity. Proc. Natl. Acad. Sci. USA.

[43] Deneweth J., Van de Peer Y., Vermeirssen V. (2022). Nearby transposable elements impact plant stress gene regulatory networks: A meta-analysis in *A. thaliana* and *S. lycopersicum*. BMC Genom..

[44] Roquis D., Robertson M., Yu L., Thieme M., Julkowska M., Bucher E. (2021). Genomic impact of stress-induced transposable element mobility in arabidopsis. Nucleic Acids Res..

[45] Wan X., Peng L., Xiong J., Li X., Wang J., Li X., Yang Y. (2019). AtSIBP1, a novel BTB domain-containing protein, positively regulates salt signaling in arabidopsis thaliana. Plants.

[46] Kim H., Kim S.H., Seo D.H., Chung S., Kim S.W., Lee J.S., Kim W.T., Lee J.H. (2016). Aba-hypersensitive BTB/POZ protein 1 functions as a negative regulator in ABA-mediated inhibition of germination in arabidopsis. Plant Mol. Biol..

[47] Liang J., Guo S., Sun B., Liu Q., Chen X., Peng H., Zhang Z., Xie Q. (2018). Constitutive expression of REL1 confers the rice response to drought stress and abscisic acid. Rice.

[48] Du H., Huang F., Wu N., Li X., Hu H., Xiong L. (2018). Integrative regulation of drought escape through ABA-dependent and -independent pathways in rice. Mol. Plant.

[49] Wei M., Zhuang Y., Li H., Li P., Huo H., Shu D., Huang W., Wang S. (2020). The cloning and characterization of hypersensitive to salt stress mutant, affected in quinolinate synthase, highlights the involvement of NAD in stress-induced accumulation of ABA and proline. Plant J..

[50] Hong Y., Wang Z., Shi H., Yao J., Liu X., Wang F., Zeng L., Xie Z., Zhu J.K. (2020). Reciprocal regulation between nicotinamide adenine dinucleotide metabolism and abscisic acid and stress response pathways in arabidopsis. PLoS Genet..

[51] Shen Z., Collatos A.R., Bibeau J.P., Furt F., Vidali L. (2012). Phylogenetic analysis of the kinesin superfamily from Physcomitrella. Front. Plant Sci..

[52] Li J., Yu D., Qanmber G., Lu L., Wang L., Zheng L., Liu Z., Wu H., Liu X., Chen Q. (2019). GhKLCR1, a kinesin light chain-related gene, induces drought-stress sensitivity in arabidopsis. Sci. China Life Sci..

[53] Kim N., Moon S.J., Min M.K., Choi E.H., Kim J.A., Koh E.Y., Yoon I., Byun M.O., Yoo S.D., Kim B.G. (2015). Functional characterization and reconstitution of ABA signaling components using transient gene expression in rice protoplasts. Front. Plant Sci..

[54] Lou D., Wang H., Liang G., Yu D. (2017). OsSAPK2 confers abscisic acid sensitivity and tolerance to drought stress in rice. Front. Plant Sci..

[55] Liu X., Li Z., Hou Y., Wang Y., Wang H., Tong X., Ao H., Zhang J. (2019). Protein interactomic analysis of SAPKs and ABA-inducible bZIPs revealed key roles of SAPK10 in rice flowering. Int. J. Mol. Sci..

[56] Sun Z., Li S., Chen W., Zhang J., Zhang L., Sun W., Wang Z. (2021). Plant dehydrins: Expression, regulatory networks, and protective roles in plants challenged by abiotic stress. Int. J. Mol. Sci..

[57] Zuo X., Cao S., Li Y., Zhang J., Ji N., Jin P., Zheng Y. (2023). Functional characterization of dehydrins CpRAB and CpERD and their roles in regulating cold resistance of zucchini fruit under high relative humidity storage. Postharvest Biol. Technol..

[58] Kumar M., Kesawat M.S., Ali A., Lee S.C., Gill S.S., Kim A.H.U. (2019). Integration of abscisic acid signaling with other signaling pathways in plant stress responses and development. Plants.

[59] Ng L.M., Melcher K., Teh B.T., Xu H.E. (2014). Abscisic acid perception and signaling: Structural mechanisms and applications. Acta Pharmacol. Sin..

[60] Tiwari P., Indoliya Y., Singh P.K., Singh P.C., Chauhan P.S., Pande V., Chakrabarty D. (2019). Role of dehydrin-fk506-binding protein complex in enhancing drought tolerance through the ABA-mediated signaling pathway. Environ. Exp. Bot..

[61] Yudina L., Sukhova E., Sherstneva O., Grinberg M., Ladeynova M., Vodeneev V., Sukhov V. (2020). Exogenous abscisic acid can influence photosynthetic processes in peas through a decrease in activity of H(+)-ATP-ase in the plasma membrane. Biology.

[62] Bharath P., Gahir S., Raghavendra A.S. (2021). Abscisic acid-induced stomatal closure: An important component of plant defense against abiotic and biotic stress. Front. Plant Sci..

[63] Sah S.K., Reddy K.R., Li J. (2016). Abscisic acid and abiotic stress tolerance in crop plants. Front. Plant Sci..

[64] Negin B., Yaaran A., Kelly G., Zait Y., Moshelion M. (2019). Mesophyll abscisic acid restrains early growth and flowering but does not directly suppress photosynthesis. Plant Physiol..

